# Linking mitochondrial and chloroplast retrograde signalling in plants

**DOI:** 10.1098/rstb.2019.0410

**Published:** 2020-05-04

**Authors:** Yan Wang, Jennifer Selinski, Chunli Mao, Yanqiao Zhu, Oliver Berkowitz, James Whelan

**Affiliations:** 1 Department of Animal, Plant and Soil Sciences, Australian Research Council Centre of Excellence in Plant Energy Biology, School of Life Sciences, La Trobe University, Bundoora, Victoria, Australia; 2 Department of Animal Science and Technology, Grassland Science, China Agricultural University, Beijing 100193, People's Republic of China

**Keywords:** mitochondria, chloroplast, retrograde signalling, alternative oxidase, common regulators

## Abstract

Retrograde signalling refers to the regulation of nuclear gene expression in response to functional changes in organelles. In plants, the two energy-converting organelles, mitochondria and chloroplasts, are tightly coordinated to balance their activities. Although our understanding of components involved in retrograde signalling has greatly increased in the last decade, studies on the regulation of the two organelle signalling pathways have been largely independent. Thus, the mechanism of how mitochondrial and chloroplastic retrograde signals are integrated is largely unknown. Here, we summarize recent findings on the function of mitochondrial signalling components and their links to chloroplast retrograde responses. From this, a picture emerges showing that the major regulators are integrators of both organellar retrograde signalling pathways.

This article is part of the theme issue ‘Retrograde signalling from endosymbiotic organelles’.

## 1. Introduction

The retrograde response pathway initiates a signalling cascade to modulate the expression of nuclear genes in response to changes in mitochondrial and chloroplastic function. In the last 30 years, our knowledge on retrograde signalling has expanded at three levels: (i) the nature of retrograde signals generated in organelles, (ii) the transducers relaying signals from organelles to the nucleus, and (iii) the transcription factors modulating nuclear gene expression (table 1). Different approaches, forward and reverse genetics, along with a range of pharmacological agents have been used to identify targets and regulators of mitochondrial signalling. While our knowledge of these areas has expanded, much still remains unknown. For instance, in the case of chloroplast retrograde signalling, it is now known that several metabolites, ranging from tetrapyrroles and various forms of reactive oxygen species (ROS) to oxidation products of carotenes such as β-cyclocitral, act as mobile signalling molecules, but the nature of the sensors leading to the production of these signalling molecules is less understood. The 3'-phosphoadenosine 5'-phosphate (PAP) phosphatase SAL1 acts as a sensor for oxidative stress in chloroplasts. SAL1 is capable of sensing changes in the photosynthetic redox poise and ROS formation (hydrogen peroxide and superoxide) via redox regulation of the enzyme that generates the signal [7]. SAL1, together with the components involved in the redox regulation of the plastid-encoded RNA polymerase [19], links photosynthetic activity with photosynthetic gene expression. However, for mitochondrial retrograde signalling, while ROS and other molecules have been implicated as signals, there is no mechanistic understanding of how these signals are transmitted to execute a response, even though downstream components have been identified [45,46]. Thus, with some exceptions such as chloroplast retrograde signalling via PAP, where the sensing [7], generation of signal [30], execution [47], integration with other pathways [48] and evolution [49] are understood at some level, there are many knowledge gaps in the various pathways that have been elucidated for both chloroplast and mitochondrial retrograde signalling.

**Table 1.** Overview of the components of retrograde signalling. Retrograde signals in organelles, the transducers relaying retrograde signalling from organelles to the nucleus and the transcription factors regulating nuclear gene expression responding to the retrograde signals are listed. Involvement of components in mitochondrial and/or chloroplast retrograde signalling is indicated with a ‘Yes’ (Y), or components implicated or under debate with a ‘question mark’ (?). For haem, as it may be synthesized in mitochondria, used as a cofactor playing a central role with tetrapyrroles in plastid retrograde signalling, and linked to retrograde signalling in other organisms, it is indicated that it may be involved in mitochondrial retrograde signalling in plants. The modulation of retrograde signalling by sugars (e.g. triose phosphate/phosphate translocator, [1]) implicates those compounds in retrograde signalling. SAL1, a 3'-phosphoadenosine 5'-phosphate (PAP) phosphatase; PAP, 3'-phosphoadenosine 5'-phosphate; MEcPP, methylerythritol cyclodiphosphate; DHAP, dihydroxyacetone phosphate; GUN1, GENOMES UNCOUPLED1; PTM, PHD TYPE TRANSCRIPTION FACTOR WITH TRANSMEMBRANE DOMAINS; PEP, plastid-encoded RNA polymerase; EX1 and EX2, EXECUTER1 and EXECUTER2; FLU, FLUORESCENT IN BLUE LIGHT; OXI1, OXIDATIVE SIGNAL INDUCIBLE1; PUB4, PLANT U-BOX 4; PRIN2, PLASTID REDOX INSENSITIVE 2; MPK3 and MPK6, MAP KINASE3 and 6; WHIRLY1, a plastid–nucleus located DNA/RNA binding protein; KIN10, SNF1 KINASE HOMOLOG 10; RS31, a serine–arginine-rich slicing factor; XRN2 and XRN3, 5′—3′ EXORIBONUCLEASE 2 and 3; STN7, STT7 HOMOLOG STN7; CDKE1, CYCLIN-DEPENDENT KINASE E1; RCD1, RADICAL-INDUCED CELL DEATH PROTEIN1; RRL, RETARDED ROOT GROWTH-LIKE protein; ABI4, ABA INSENSITIVE4; ANAC017 and ANAC013, ARABIDOPSIS NAC DOMAIN CONTAINING PROTEIN 17; MYB29, MYB DOMAIN PROTEIN29; WRKY40, WRKY DOMAIN PROTEIN40; WRKY63, WRKY DOMAIN PROTEIN63; WRKY15, WRKY DOMAIN PROTEIN15; GLK1 and GLK2, GOLDEN2-LIKE 1 and 2.

component of retrograde signalling	mitochondria	chloroplasts	reference
*signals in organelles*			
hydrogen peroxide (H_2_O_2_)	?	Y	[2,3]
singlet oxygen (^1^O_2_)	?	Y	[2,4,5]
SAL1- PAP	Y	Y	[6,7]
haem	?	Y	[8,9]
β-cyclocitral		Y	[5]
MEcPP		Y	[10]
DHAP		Y	[1]
salicylic acid	Y	Y	[11,12]
calcium	Y	Y	[13,14]
*signal transduction (from organelles to nucleus)*			
GUN1		Y	[15,16]
PTM		?	[17,18]
PEP		Y	[19,20]
EX1, EX2		Y	[21,22]
FLU		Y	[23]
OXI1		Y	[24]
PUB4		Y	[25]
PRIN2		Y	[19]
MPK3, MPK6		Y	[1,14]
WHIRLY1		Y	[26]
KIN10	Y	Y	[27,28]
RS31		Y	[29]
XRN2, XRN3	?	Y	[30]
STN7		Y	[31]
CDKE1	Y	Y	[27,32]
RCD1	Y	Y	[33]
RRL	Y	?	[34]
sugars	?	?	[1,35]
*transcription factor*			
ABI4	Y	?	[15,36,37]
ANAC017	Y	Y	[38–40]
ANAC013	Y	Y	[39,41]
AP2/ERF-TFs		Y	[1]
MYB29	Y		[42]
WRKY40/63	Y	Y	[43]
WRKY15	Y		[13]
GLK1, GLK2		Y	[44]

The alternative oxidase (AOX) is by far the most commonly used indicator to study mitochondrial retrograde signalling [45,50,51]. AOX is a cyanide-insensitive terminal oxidase in mitochondria that increases in abundance at a transcript and protein level in response to a variety of perturbations, including genetic mutations, nutrient availability and oxidative stresses [52–56]. In *Arabidopsis*, *AOX1a* has been used as a key marker of mitochondrial retrograde regulation, since the transcript abundance can be induced in response to mitochondrial stresses caused by antimycin A (AA) or monofluoroacetate (MFA), which inhibit mitochondrial electron transport and the tricarboxylic acid (TCA) cycle, respectively [57,58]. However, AA also inhibits photosynthetic electron transport [59–61]. This suggests that a retrograde signal triggered by the inhibition of chloroplastidic electron transport activity also contributes to the induction of *AOX1a* [32], and it has been shown that AOX plays a crucial role in maintaining photosynthesis under high light or drought conditions [62,63]. One model suggests that this protection at least in part comes from maintaining photorespiration and export of reducing equivalents from chloroplasts by the malate/oxaloacetate shuttle [64]. As a variety of other studies showed that AOX expression is also increased by high light stress and genetic lesions affecting chloroplast activity [15,36], it needs to be noted that AOX is induced by both mitochondrial and chloroplast perturbation. Thus, additional models for mitochondrial signalling responding specifically to mitochondrial perturbation need to be developed. One potential candidate is the *At12Cys-2* (At5g09570) gene, for which transcript abundance is induced in a variety of genetic mutants in response to chemical treatments impacting mitochondrial (but not chloroplast) function and a variety of abiotic stresses. In fact, it is more responsive than the induction of *AOX1a* in many instances [65]. However, so far, the induction of At12Cys-2 protein level is only observed in the mutants with decreased respiratory complex I activity [65], indicating that the abundance of At12Cys-2 at a protein level is post-transcriptionally regulated in response to a specific mitochondrial lesion(s) in complex I, while for AOX1a, an increase in protein abundance always accompanies an increase in transcript abundance following stimulation [27,38]. As complex I is the entry point of electrons into the electron transport chain, a specific signalling pathway sensitive to changes in complex I activity or dysfunction could control both cytochrome and alternative respiratory pathways. Another protein that has been used to analyse mitochondrial stress-induced proteins is the outer mitochondrial membrane protein 66 (OM66). It is induced at a transcript and protein level by different pathways similar to *AOX* [66], but it is yet unclear if these signals are strictly retrograde, i.e. generated in mitochondria (see below).

The signals, transducers and effectors of chloroplast retrograde signalling are more widely studied and better characterized than those for mitochondria [67]. Chloroplast retrograde signalling has been classified into biogenic and operational levels. Biogenic retrograde signalling occurs during plastid development, most notably when plants develop from being heterotrophic to autotrophic with signals from the developing chloroplast coordinating nuclear gene expression. Operational retrograde signalling optimizes organelle function with environmental conditions. Studies on both levels identified signals (e.g. metabolites, various ROS, transducers and effectors (e.g. hypocotyl 5 (HY5), heat shock transcription factors, 5′–3′ exoribonucleases (XRNs)) [7,68,69]. It is beyond this review to describe all the components of chloroplast retrograde signalling in detail. Therefore, we will focus on the identification of overlaps between chloroplast and mitochondrial retrograde signalling, both being stimulated by either the same signals or component (e.g. translation) or shared components involved in transduction or execution (e.g. SAL1). While mitochondrial retrograde signalling could also be classified into biogenic and operational levels, to date all the studies on mitochondrial retrograde signalling would be classified as operational, as they are carried out in response to internal or external stimuli during vegetative growth. While it would be extremely interesting to study, mitochondrial biogenic control, unlike photosynthetic function mitochondrial function, is extremely important for seed germination, and a ‘burst’ of mitochondrial biogenesis does take place as one of the earliest events in germination [70,71]. In fact, many mitochondrial proteins are encoded by small gene families where some isogenes display preferential expression at a specific stage of germination [72], and mutations of these genes often result in greatly altered seedling morphology or lethality [70]. Thus, to our knowledge, no studies have investigated retrograde signalling during mitochondrial biogenesis.

Analyses of transcriptome signatures for mitochondrial and chloroplastic responses revealed a variety of induced genes encoding proteins localized not only to mitochondria or chloroplasts but also to other cell compartments [73]. This is not surprising as alterations of mitochondrial or chloroplast function will affect the whole plant cell. Given this global impact and the metabolic connectivity between mitochondria and chloroplasts, there is a need to coordinate the function between these organelles, and retrograde signalling is a major means to maintain cellular homeostasis. Thus, it is expected that there will be shared components between mitochondrial and chloroplast retrograde signalling pathways (table 1).

## 2. Regulators of mitochondrial retrograde signalling

### (a) Regulators of *AOX1a* retrograde signalling

As outlined above, AOX has been widely used as a model for mitochondrial retrograde signalling, and therefore it is not surprising that it is the best-characterized target for the identification of regulators of nuclear genes encoding mitochondrial proteins. Pharmacological studies primarily using *Nicotiana tabacum* (tobacco) and *Arabidopsis* suggest that both ROS and non-ROS pathways exist to induce AOX expression. For the ROS-mediated pathway, inhibition of the cytochrome electron transport chain with inhibitors such as AA induces the generation of ROS as determined by the induction of fluorescent signals co-locating with mitochondria, and overexpression of AOX suppresses the induction of ROS after inhibition by AA [74]. Thus, AOX acts as a pre-oxidant defence system, preventing the production of ROS when electron flow via the cytochrome electron transport chain is restricted. The non-ROS pathway is also best characterized in tobacco, where citrate is a potent inducer of AOX [75,76]. However, in both *Arabidopsis* and soya bean (*Glycine max*), the extent of induction by citrate is more limited [77,78]. The induction of *AOX1a* in *Arabidopsis* under a variety of conditions such as nutrient limitation and the expression of other isoforms such as *AOX1c* suggest that non-ROS pathways may also exist [56,79], but corresponding signals and components have not yet been identified.

**Figure 1.**
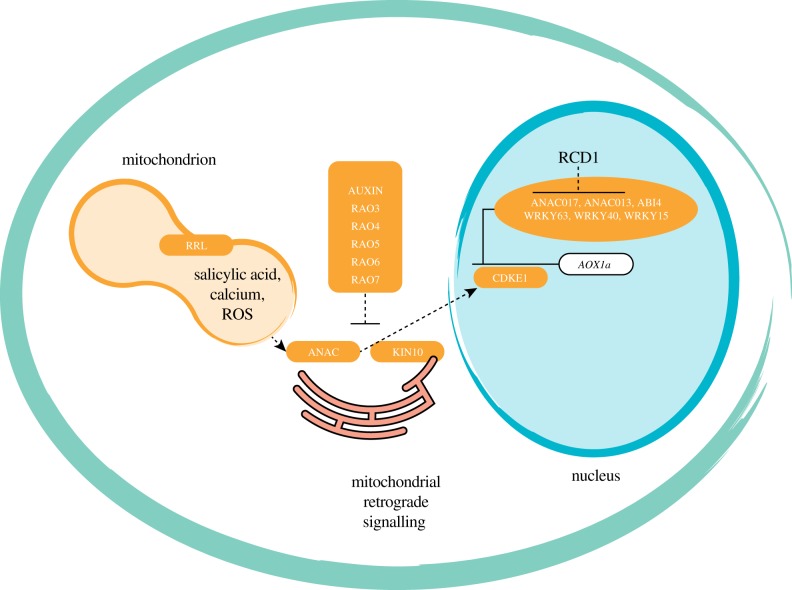
Retrograde regulation of *AOX1a.* A number of components that regulate the expression of AOX1a have been identified. Upon mitochondrial dysfunction, activation of a number of ER-bound ANAC transcription factors occurs, with ANAC017 being the master regulator regulating the expression of a number of other transcription factors. Other positive regulators identified include WRKY63 and ANAC013. CDKE1, a subunit of the kinase module of the Mediator complex, was also shown to be required for the induction of *AOX1a*, and interacts with KIN10. The latter has been shown to dynamically move between the ER and the nucleus. A number of negative regulators, including ABI4, WRKY40 and WRKY15, have also been identified. Other negative regulators include MYB29, components involved in auxin signalling (RAO3, 4, 5, 6 and 7) and RCD1. Finally, it has been shown that a dual-targeted protein, called RETARDED ROOT LIKE (RRL), is required for the translation of *AOX1a* and accumulation of AOX1a. ROS, reactive oxygen species; RRL, RETARDED ROOT GROWTH-LIKE protein; RAO, Regulator of Alternative Oxidase 1A; ANAC, the membrane-bound NAC transcription factors; KIN10, SNF1-related protein kinase; CDKE1, CYCLIN-DEPENDENT KINASE E1; RCD1, RADICAL-INDUCED CELL DEATH PROTEIN 1; ABI4, ABA INSENSITIVE 4; WRKY63, WRKY DOMAIN PROTEIN 63; WRKY40, WRKY DOMAIN PROTEIN 40; WRKY15, WRKY DOMAIN PROTEIN 15; AOX, Alternative Oxidase. (Online version in colour.)

A forward genetic screen to identify regulators of *AOX1a* in *Arabidopsis* identified the NAC transcription factor ANAC017 (RAO2, REGULATOR OF AOX1A 2) as a master regulator of mitochondrial retrograde signalling [38] (figure 1). A latent form of this transcription factor is present at the endoplasmic reticulum (ER), and upon cleavage by a rhomboid protease it is translocated to the nucleus, where it reprogrammes retrograde stress responses comprising several hundred genes. These include downstream transcription factors of the WRKY and ANAC families as well as BASIC LEUCINE-ZIPPER MOTIF transcription factors involved in the ER unfolded protein response and the balancing of energy homeostasis via the SNF1-RELATED PROTEIN KINASE (KIN10) [40]. Other regulators identified in this screen include CYCLIN-DEPENDENT KINASE E1 (CDKE1, RAO1), a subunit of the RNA Mediator complex, the transcription factor MYB29 (RAO7) and several components involved in auxin signalling (RAO3, RAO4, RAO5 and RAO6) [27,42,80] (figure 1). The involvement of CDKE1 and its interaction with KIN10, a central mediator of stress and energy signalling in the cell [81], is likely to be involved in the non-ROS signalling pathway and links mitochondrial retrograde signalling to overall cellular energy signalling (figure 1). MYB29, a regulator of aliphatic glucosinolate synthesis [82], and several components involved in auxin signalling are negative regulators of mitochondrial signalling, required to shut down the process (figure 1). The facts that ANAC017 is not inducible at a transcript level and is present in a latent form [38], overexpression of ANAC017 results in early senescence [40], the existence of several negative regulators of *AOX1a* [43] and the recent demonstration that RADICAL-INDUCED CELL DEATH PROTEIN1 (RCD1) binds to ANAC017 to suppress its activity [33] (figure 1) together show that while mitochondrial retrograde signalling is important for environmental stress responses, it is kept highly suppressed under non-limiting growth conditions.

Reverse genetic approaches have also been successful in the identification of components regulating *Arabidopsis* *AOX1a*. Transcriptome meta-analysis identified the mitochondrial dysfunction motif (MDM) of several mitochondrial dysfunction stimulon (MDS) genes, including *AOX1a*, *UPOX* and *At12Cys-2*. A yeast-one-hybrid screen identified the NAC transcription factor ANAC013 as a regulator of mitochondrial retrograde signalling [41] (figure 1). Direct binding of ANAC017 to the MDM present in the ANAC013 promoter suggests that ANAC013 is regulated by ANAC017 [41]. This is consistent with the fact that ANAC017 was identified by a forward genetic screen showing that ANAC013 could not compensate for inactivation of ANAC017. Additionally, *ANAC013* was upregulated in transcript abundance in overexpression lines of ANAC017 [40]. A similar approach using transcriptome meta-analysis combined with yeast-one-hybrid assays demonstrated the binding of 12 WRKY transcription factors in the promoter regions of *AOX1a*, *NDB2* and *OM66* and their function as possible regulators of mitochondrial retrograde signalling [43]. WRKY40 was identified to be a repressor of *AOX1a* retrograde signalling (figure 1). After AA treatment or high light stress, a significantly higher induction of *AOX1a* was observed in *wrky40* knockout plants compared with the wild-type, while less induction was observed in *WRKY40* overexpression lines [43]. WRKY63 was shown to be an activator of *AOX1a*, as a significantly higher induction of *AOX1a* was observed in *WRKY63* overexpression lines after high light stress compared with the wild-type [43] (figure 1). Transcriptome-wide analysis revealed that WRKY40 and WRKY63 were involved in regulating the expression of genes responding to both mitochondrial and chloroplast dysfunction, but not genes responding to mitochondrial or chloroplast dysfunction alone [43]. This suggests a role of WRKY40 and WRKY63 in coordination of mitochondrial and chloroplast function through retrograde signalling. Another possible repressor regulating *AOX1a* expression is WRKY15 (figure 1). The induction of *AOX1a* and mitochondrial dysfunction regulon (MDR) genes after salt stress application was inhibited in *WRKY15* overexpression plants [13]. It was proposed that calcium flux sensing might be triggering the mitochondrial retrograde cascade through an interaction with the Ca^2+^-dependent CaM-binding domain of WRKY15 [13].

ABA INSENSITIVE 4 (ABI4) was the first identified regulator of *AOX1a* in *Arabidopsis* (figure 1). It has been shown that *AOX1a* expression is highly induced in *abi4* mutant plants and ABI4 binds to the *AOX1a* promoter [36], which suggests ABI4 acts as a repressor of *AOX1a* under normal conditions. External addition of abscisic acid (ABA), AA and rotenone, respectively, can lift this repression. It has been reported that the induction of both *AOX1a* and *ABI4* was inhibited after ABA treatment in the knockout plants of RETARDED ROOT GROWTH-LIKE (RRL), leading to the conclusion that RRL plays a role upstream of ABI4 to regulate the expression of *AOX1a* after ABA treatment [34]. However, subsequent studies revealed that RRL is a dual-targeted protein that is localized to mitochondria and chloroplasts, and that hyper-induction of *AOX1a* and other mitochondrial stress responsive genes occurs in *rrl* mutants at a transcript level, but an induction at the protein level is suppressed [83]. Thus, RRL may represent a novel component of mitochondrial retrograde signalling required for regulation at the post-transcriptional level.

### (b) *OM66* retrograde signalling

*OM66* is one of 26 genes encoding mitochondrial proteins whose transcript abundance is highly stress inducible [11,84]. This gene encodes a mitochondrial outer-membrane protein present in a homo-multimeric protein complex. The promoter of *OM66* is highly responsive to salicylic acid (SA), unlike the promoter of *AOX1a*, which is responsive to H_2_O_2_ and rotenone [11]. Analysis of *OM66* transcript abundance in mutants compromised in a variety of defence signalling pathways reveals that *OM66* expression is regulated in a manner distinct from *AOX1a*, but follows the pattern of the positive regulator of SA responses *PATHOGEN-RELATED PROTEIN 1* (*PR1*) [11]. The expression of *PR1* was reduced in *om66* mutants and a higher SA content was observed in *OM66* overexpression lines [11]. This suggests that OM66 is regulated by an SA-dependent signalling pathway with a number of WRKY binding sites present in the promoter of *OM66* [43]. WRKY63 was shown to affect the basal expression of *OM66* as its transcript abundance was highly induced in *WRKY63* overexpression lines and significantly reduced in *wrky63* knockout plants under non-limiting growth conditions, which was not observed for *AOX1a* expression [43]. While WRKY40 did not affect basal expression, it acted as a repressor of *OM66* in the stress response to AA and high light treatment [43]. It is not yet clear if the increase in *OM66* transcript abundance is primarily a response to a retrograde signal generated in mitochondria. *OM66* transcript abundance significantly increase after 3 h of rotenone or AA treatment [11,66]. However, as the transcript abundance also increases upon flagellin or touch treatment within 30 min [66,85,86] and its expression is *nahg* (salicylate hydroxylase)-dependent but *npr1* (Nonexpressor of PR-1)-independent [11], it cannot yet be concluded if *OM66* is a direct target of mitochondrial retrograde signalling or its responses are due to perturbation of mitochondrial function. Several reports show an interaction between mitochondrial function and SA, including: (i) SA inhibits respiration through both the cytochrome and alternative respiratory pathways, and selectively accumulates in mitochondria [87,88], (ii) SA binds to and inhibits the E2 subunit of α-ketoglutarate dehydrogenase, a TCA cycle enzyme [89], and (iii) mitochondrial and SA signalling are linked [88,90,91]. Given the potential role of SA in chloroplast retrograde signalling evidenced by the accumulation of SA upon damaging of photosystem II reaction centre proteins [12], SA may also connect mitochondrial and chloroplast retrograde signalling. It is notable that *OM66* does have the MDM and that the transcript abundance of *OM66* is affected in both over-expressing and knockout lines of *ANAC017/RAO2* [40,92]. Thus, given the rapid induction after touch or application of flagellin, the response to mitochondrial perturbation may be secondary, and altered *OM66* transcript abundance in *ANAC017/RAO2* mutant and overexpression lines may be due to the greatly altered expression of a wide variety of other transcription factors and other processes such as growth and senescence [40].

### (c) *At12Cys-2* retrograde signalling

At12Cys-2 belongs to a protein family defined by two pairs of cysteine residues each separated by nine amino acids (CX_9_C) [93]. The members in *Homo sapiens* are known to be subunits of complex I [94,95], and several protein family members in yeast are involved in the assembly of cytochrome oxidase [96,97]. In *Arabidopsis*, *At12Cys-2* transcript abundance was increased in a wide variety of mitochondrial perturbations, but importantly the protein level was only induced in the mutants with compromised mitochondrial complex I activity [65]. The At12Cys-2 protein was shown to co-migrate with supercomplex I + III on BN-PAGE (Blue Native PAGE) and the activity of complex I was reduced in *at12cys-2* mutants [65]. More interestingly, the At12Cys-2 protein was found in mitochondria, cytosol and chloroplasts in mutants with reduced complex I activity, while it was only located in mitochondria in wild-type plants [65]. Alterations of the At12Cys-2 protein level also disturbed the abundance of mitochondrial, cytosolic and chloroplast proteins [65]. Thus, *At12Cys-2* is an ideal marker gene for mitochondrial stress retrograde signalling, and furthermore, it may play a central role in relaying complex I deficiency stress to outside the mitochondria, mediating cell-wide responses.

So far, little is known about the regulators involved in *At12Cys-2*-dependent retrograde signalling. The ANAC013 transcription factor binds to the promoter of *At12Cys-2* [41]. The transcript of *At12Cys-2* was highly induced in *ANAC013* overexpression lines and the induction after AA treatment was dramatically diminished in lines with reduced ANAC013 expression (*ANAC013-*miR) [41]. These results indicate that ANAC013 is a positive regulator of the *At12Cys-2*-dependent retrograde signalling pathway. That upregulation of *At12Cys*-*2* transcripts in mutants with disrupted mitochondrial function does not always lead to concomitant increases at protein levels, it suggests that additional factors are required for the induction of At12Cys-2 post-transcriptionally. One possible candidate is RRL, which is required for the increase in protein abundance downstream of mitochondrial signalling ([83]).

## 3. Common regulators of mitochondrial and chloroplast retrograde signalling

Given the interaction in metabolism between chloroplasts and mitochondria, it is not surprising that signalling pathways from these organelles may share several components. In addition to metabolic interactions, chloroplasts and mitochondria also share many proteins, so-called dual-targeted proteins, where the same protein is targeted to both organelles. In fact, one elegant example of the coordination and integration of signalling from chloroplasts and mitochondria was observed with a dual-targeted protein, prolyl-tRNA synthetase [98]. This study revealed that signals from both chloroplasts and mitochondria cooperate or have a synergistic effect to alter nuclear gene expression. Given that over 100 dual-targeted proteins have been characterized [99], and as many as 400 have been predicted to be dual targeted [100], integrated regulation of the expression of these proteins is likely.

### (a) ABI4

ABI4 was initially identified as a repressor of seed germination [101]. In the last 20 years, it has been shown to play a variety of roles in flowering [102], light signalling [14], biotic stresses in response to spider mite resistance [103], and abiotic stresses such as drought and salt [104,105], and it is involved in the interaction between sugar, hormone and redox signalling [106]. In 2007 and 2009, two separate reports linked ABI4 to plastid and mitochondrial retrograde signalling, acting as a repressor of the light-harvesting chlorophyll *a*/*b* binding protein (*Lhcb*) and *AOX1a*, respectively [15,36]. For mitochondrial signalling, the depression of *AOX1a* transcription in *abi4* mutant backgrounds, the ability of ABI4 to bind to a consensus motif shown with both EMSA and yeast-1-hybrid assays, and the deletion of this motif resulting in constitutive activation of the *AOX1a* promoter provide direct evidence for the role of ABI4 in regulating the expression of *AOX1a* [36]. However, application of ABA induces the expression of *AOX1a*, which means that either ABI4 can be both a positive and negative regulator, as has been described [36], or that other ABA-responsive factors can bind.

**Figure 2.**
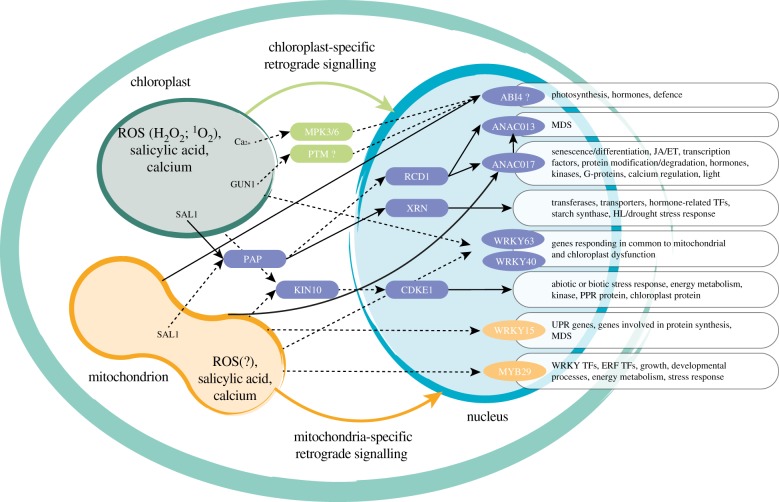
Common regulators of mitochondrial and chloroplast retrograde signalling. A number of components playing a role in both mitochondrial and chloroplast retrograde signalling have been reported. SAL1, accumulating in both organelles, activates retrograde signals via PAP and XRNs which regulate nuclear gene expression through RNA processing or mRNA decay. The SAL1/PAP pathway is also proposed to overlap with RCD1, which interacts with the transcription factors ANAC013 and ANAC017. WRKY63 and WRKY40 regulate genes common in response to mitochondrial and chloroplast dysfunction but as yet signals or components leading to their activation have not been characterized. KIN10, having the potential to sense the retrograde signals from both organelles, relays the information to nuclear-located CDKE1. Orange and green colours indicate components specific to mitochondrial and chloroplast retrograde signalling, respectively. Components that are putatively shared between mitochondrial and chloroplast retrograde signalling are indicated in purple. Where a role has been experimentally shown, it is indicated with a solid line. Roles that are proposed on the basis of changes of transcript abundance alone or questioned are indicated with dashed lines. ROS, reactive oxygen species; MPK3 and MPK6, MAP kinase 3 and 6; GUN1, GENOMES UNCOUPLED 1; PTM, a chloroplast envelope-bound PHD transcription factor; SAL1, phosphatase-like protein; PAP, 3'-phosphoadenosine 5'-phosphate; KIN10, SNF1-related protein kinase; XRN, 5′–3′ exoribonuclease; RCD1, RADICAL-INDUCED CELL DEATH PROTEIN 1; CDKE1, CYCLIN-DEPENDENT KINASE E1; ABI4, ABA INSENSITIVE 4; ANAC017 and ANAC013, the membrane-bound NAC transcription factors; WRKY40, WRKY DOMAIN PROTEIN 40; WRKY63, WRKY DOMAIN PROTEIN 63; WRKY15, WRKY DOMAIN PROTEIN 15; MYB29, MYB DOMAIN PROTEIN 29; MDS, mitochondrial dysfunction stimulon genes; JA/ET, jasmonic acid/ethylene; TF, transcription factor; HL, high light; PPR, pentatricopeptide repeat; UPR, unfolded protein response; ERF, ETHYLENE RESPONSE FACTOR. (Online version in colour.)

For plastid gene expression, ABI4 was initially identified as a nuclear component of GUN1-mediated chloroplast retrograde signalling (figure 2); however, its role in biogenic retrograde signalling has been questioned recently [37]. GUN1 is a chloroplast pentatricopeptide-repeat (PPR) protein. The inactivation of GUN1 was shown to depress the induction of photosynthesis-associated nuclear genes (PhANGs), such as the gene for a light-harvesting chlorophyll *a*/*b* binding protein (Lhcb), in multiple chloroplast retrograde pathways, including tetrapyrrole-mediated, photosynthetic electron transport (PET)-dependent and plastid gene expression (PGE)-mediated pathways [16]. While the role of GUN1 in chloroplast biogenic retrograde signalling was verified by several groups [37], the role of either ABI4 or a chloroplast envelope-bound PHD transcription factor (PTM) could not be confirmed in other laboratories. The derepression of several genes, such as *LHCB1.2, LHCB2.1 HEMA1, CHLH, GUN4, CARBONIC ANHYDRASE1 (CA1)* [107] or *GOLDEN2-LIKE1 (GLK1)* [108], after treatment with lincomycin and norflurazon as the hallmark of the *gun* phenotype was not observed in *abi4* mutant backgrounds [37]. Secondly, transcriptome re-analysis does not support that *abi4* mutants respond in a similar manner to *gun* mutants as was originally suggested [15], with *abi4* clustering with wild-type plants after lincomycin treatment [37]. Furthermore, the MAP kinases MPK3 and MPK6 have also been proposed as transducers relaying retrograde signals from chloroplasts to ABI4 (figure 2) [14]. Given that multiple laboratories are questioning the role of ABI4 and PTM, the role of MAP kinases now also needs to be re-evaluated. While the current consensus that ABI4 and proposed interacting components are not involved in biogenic chloroplast retrograde signalling, it would be productive if the discrepancies between the studies can be explained. A cautionary example is observed with LOW PHOSPHATE RESPONSE1 (LPR1) or 2 (LPR2). Mutation in LPR1 or LPR2 resulted in the insensitivity of *Arabidopsis* primary root growth to P_i_ deficiency [109]; however, these results could not be reproduced in some laboratories. Such discrepancies in observations were puzzling, given the perceived identical growth conditions, but a recent report showed that blue-light-mediated ROS production in roots of plants grown on Petri dishes, a variable of lighting conditions in growth chambers, can cause this phenotype [110]. Thus sometimes, unknown variables that are quite small may have a large impact on the interpretation of results.

### (b) SAL1–PAP and RCD1 convergent with ANAC

The SAL1–PAP-mediated pathway is one of the best-characterized chloroplast retrograde signalling pathways [7]. SAL1 is a bifunctional protein that has 3'-phosphoadenosine 5'-phosphate (PAP) phosphatase and inositol polyphosphate 1-phosphatase activities and is located in both mitochondria and chloroplasts [6]. The phosphatase activity of SAL1 is redox-regulated by intramolecular disulfide bond formation, dimerization and glutathionylation in response to changes in ROS concentrations and photosynthetic redox poise in chloroplasts [6,7]. SAL1 regulates the level of 3′-phosphoadenosine 5′-phosphate (PAP) by dephosphorylating PAP to AMP, which is supported by the increased PAP level in *sal1* mutants [6]. In addition, the activity of PAPS TRANSPORTER2, localized to plastidial and mitochondrial membranes, controls the accumulation of PAP in the cytosol [111]. The movement of PAP between the nucleus and chloroplasts was demonstrated by restoring the PAP content in *sal1* mutants using either chloroplast- or nucleus-targeted SAL1 protein [6]. PAP functions as a mobile transducer relaying the retrograde signals from chloroplasts to the nucleus. In the nucleus, PAP was proposed to affect the activity of 5′–3′ exoribonucleases (XRNs), as demonstrated in yeast [112], leading to changes in nuclear gene expression [47]. In *Arabidopsis*, three genes encode XRNs: the nuclear-localized XRN2/XRN3 and the cytosolic XRN4 [113]. These proteins play important roles in RNA processing and mRNA degradation [113,114]. The transcript profiles of *xrn2xrn3* mutants had a significant overlap with those of a PAP-accumulating mutant and increased levels of 3′-mRNA cleavage products were observed in the mutants deficient in SAL1 or XRN2/XRN3, in support of nuclear XRNs being targets of the SAL1–PAP retrograde pathways [6]. The mechanism how XRNs mediate gene expression was recently demonstrated. RNA polymerase II (RNA Pol II) 3′ read-through was the major consequence of reducing the activity of SAL1 or XRNs, and XRN2/XRN3 are required for RNA Pol II termination [47]. RNA Pol II read-through due to the reduced activity of SAL1 or XRN2/XRN3 further upregulates the transcript levels of downstream genes [47]. There is no strong evidence showing that XRN4 is involved in the SAL1–PAP retrograde pathway.

The validation of SAL1 dual-targeting to chloroplasts and mitochondria and PAP accumulating in both organelles suggest that SAL1 and PAP also play an important role in mitochondrial retrograde signalling (figure 2). One study proposed that the SAL1–PAP retrograde signalling pathway is likely convergent with the ANAC017-mediated mitochondrial retrograde pathway, based on the observation that the target genes of ANAC017- and PAP-dependent signalling pathways are partially overlapping [39]. This hypothesis is supported by another independent study, showing that the SAL1–PAP-signalling pathway intersects with the RCD1-dependent pathway [33]. RCD1 is a nuclear protein whose abundance, thiol redox state and oligomerization are affected by ROS production in chloroplasts [33]. The *rcd1* mutation compromised the response to chloroplast ROS and also changed mitochondrial AOX respiration and energy metabolism [33]. The genes mis-regulated in the *rcd1* mutant had a significant overlap with the genes affected by the PAP-signalling pathway and the mitochondrial dysfunction stimulon genes, including those for AOX1a and the sulfotransferase SOT12, an enzyme generating PAP [33]. The *sal1* and *rcd1* double mutant displayed a more severely retarded growth phenotype than the single mutants [33]. These results suggest that the RCD1-dependent retrograde pathway overlaps or converges with the SAL1–PAP-dependent pathway. Furthermore, RCD1 was demonstrated to interact with ANAC transcription factors in a yeast-two-hybrid assay and two independent pull-down assays and it was suggested to act as a negative regulator of ANAC013 and ANAC017 [33]. The *rcd1* and *anac017* double-mutant plants are more sensitive to chloroplast ROS than the *rcd1* single mutant. Also, the AOX abundance/respiration capacity was much lower in the double mutant than the *rcd1* mutant, indicating that ANAC017 could mediate both chloroplast- and mitochondria-derived retrograde signals, with RCD1 acting as a negative regulator [33]. Together, these results provide evidence for a role of RCD1 in integrating ROS signals from both mitochondria and chloroplasts and modulating nuclear gene expression through the regulation of transcription factors, including ANAC013 and ANAC017.

### (c) CDKE1

While CDKE1 was first identified as an essential component of mitochondrial retrograde signalling in *Arabidopsis* in response to inhibitors affecting mitochondrial function, it is also required for the regulation of *AOX1a* in response to more general cellular oxidative stresses such as H_2_O_2_ treatment and cold stress [27]. A role in chloroplast operational retrograde signalling is supported by another study, showing that CDKE1 regulates the expression of *AOX1a* and *Lhcb2.4* in response to 3-(3,4-dichlorophenyl)-1,1-dimethylurea (DCMU) and 2,5-dibromo-3-methyl-6-isopropyl-benzoquinone (DBMIB), which are inhibitors exclusively affecting the photosynthetic electron transport chain [32]. Under high light stress, the *cdke1* mutants displayed impaired ability to recover photosystem efficiency and suffered a growth penalty during the initial stages of heterotrophic growth, which was similar to the *gun* phenotype upon induction of redox stress originating from chloroplast electron transport [32]. Based on these two independent studies, it is suggested that CDKE1 integrates the retrograde signals generated in both mitochondria and chloroplasts to modulate nuclear gene expression [68].

## 4. Conclusion and future perspectives

The biochemical, cellular and physiological reasons why mitochondrial and chloroplast retrograde signalling may be linked at several levels are worth considering. Organelle retrograde signalling provides feedback to anterograde signals that alters gene expression encoding proteins located in a variety of locations in the cell. Thus, chloroplasts and mitochondria have emerged as environmental sensors to ensure that the pipeline from gene expression to protein function is optimized relative to organelle function. An integral function of mitochondria and chloroplasts in energy biology in plants means that any functional perturbation in either organelle will have cellular and plant-wide consequences. Thus, signals emanating from organelles alongside their essential central roles in energy biology would be an efficient means to ensure whole cellular function is tuned to the functional status of these organelles. Retrograde signalling occurring early in the evolution of land plants [115] and in mitochondria from single-celled yeast and *Caenorhabditis elegans* [116], and the evidence of retrograde signalling in *Chlamydomonas reinhardtii* [117] indicate that it is likely essential in single-celled aquatic algae. Thus, given the evolutionary time spans involved, it is not surprising that chloroplastidic and mitochondrial pathways have merged or shared components.

The coordination of chloroplast and mitochondrial retrograde signalling provides plants with a mechanism to maintain energy homeostasis in cells [32]. Given the central role of both organelles in primary and secondary carbon and nitrogen metabolism, along with essential biosynthetic functions required for growth, the functions of the two organelles must be coordinated. This coordination can be achieved at a variety of levels involving metabolite transport and exchange between organelles, but can also occur at the level of gene expression. Operational retrograde control optimizes organelle function with environmental conditions, and thus, given the metabolic interdependence, it is not surprising that the retrograde signalling may be coordinated by shared regulators. As an example, the involvement of CDKE1 in chloroplast and mitochondrial retrograde signalling positions it to relay signals to activate stress-induced gene expression sensed by both mitochondria and chloroplasts, switching between growth and stress responses via the Mediator complex [68]. Chloroplast and mitochondria also interact with several hormone signalling pathways. ABA, jasmonic acid and SA are all synthesized in chloroplasts and interact with operational chloroplast retrograde signalling [48,118], while application of ABA induces *AOX1a* and SA inhibits mitochondrial respiration via both the cytochrome and alternative pathways [36,87,119]. Independent studies have shown that mitochondrial signalling and auxin signalling are antagonistic and also that the role of the conserved chloroplast metabolite signalling molecule MEcPP (methylerythritol cyclodiphosphate) in modifying auxin signalling and transport [120] provides another convergence point for organelle signalling. Thus, it is likely that future studies will reveal even more interaction and common signals, transducers and executers of chloroplast and mitochondrial retrograde signalling.

It has been reported that GUN1 regulates chloroplast protein import through interaction with chloroplast HSP70 during chloroplast biogenesis and under adverse conditions [121]. The inhibition of protein import leads to upregulation of the cytosolic machinery to degrade unimported chloroplast precursor proteins. It has also been shown that the ubiquitin E3 ligase SP1 with a multicomponent system is involved in the degradation of outer envelope proteins [122]. In a similar manner to the involvement of the cytosolic proteasome system in regulating chloroplast retrograde signalling, the ubiquitin-mediated degradation of mitochondrial protein and/or of retrograde signalling components has been observed in yeast [123,124]. As *Arabidopsis* SP1 has also been reported to be located in mitochondria and peroxisomes [125], albeit there is some discussion about the multiple locations of SP1 in plants [126], it would be an elegant system to coordinate the biogenesis and function of chloroplasts, mitochondria and peroxisomes. Thus, studies investigating the retro-translocon of mitochondrial proteins to the cytosol and the role of the cytosolic proteasome system in regulating mitochondrial biogenesis may provide another common point for regulation of chloroplast and mitochondrial retrograde signalling.

For future studies on identifying links between chloroplast and mitochondrial retrograde signalling, it will be important to define what constitutes a common component rather than where pathways converge to a common point to interact with other signalling pathways. While elucidation of these common convergence points is informative, this is not exclusive to retrograde pathways. From figure 2, it is clear that there are many gaps in our knowledge of retrograde pathways, including about the generation of the initial signals, the transducers and the endpoint execution. Although for the latter some regulators of retrograde-regulated genes are identified (e.g. WRKY transcription factors), the upstream signalling events are still yet unknown. One possible fruitful approach to defining more links between chloroplast and mitochondrial retrograde pathways will be to investigate the regulation of dual-targeted proteins in more detail. In addition to prolyl-tRNA synthethase, which represents a protein that is dual-targeted to chloroplasts and mitochondria as outlined above, over 100 dual-targeted proteins have been defined in *Arabidopsis*, and from predictions several hundred may exist. The activation of dual-targeted protein expression would be an ideal way to coordinate organelle function. As dual-targeted proteins are involved in organellar transcription, translation, proteolysis, anti-oxidant defence and metabolism, it appears they could impact many of the pathways known to activate retrograde signalling. Both the RRL and At12Cys proteins discussed above are located in both chloroplasts and mitochondria, and while their role(s) in organelle retrograde signalling are not known, they are likely to coordinate some functions that will link chloroplast and mitochondrial signalling. Finally, with the emergence of the ability to generate cell-specific transcriptomes and even the possibility of cell-specific proteomes [127], the analysis of cell-specific organellar signalling will become possible and a first example is the identification of tissue-specific sensory plastids [128].

## Data accessibility

This article has no additional data.
